# Adipokine Contribution to the Pathogenesis of Osteoarthritis

**DOI:** 10.1155/2017/5468023

**Published:** 2017-04-08

**Authors:** Daniel Azamar-Llamas, Gabriela Hernández-Molina, Bárbara Ramos-Ávalos, Janette Furuzawa-Carballeda

**Affiliations:** Department of Immunology and Rheumatology, Instituto Nacional de Ciencias Médicas y Nutrición, Salvador Zubirán, Vasco de Quiroga No. 15, Col. Belisario Domínguez, Sección XVI, 14080 Mexico City, Mexico

## Abstract

Recent studies have shown that overweight and obesity play an important role in the development of osteoarthritis (OA). However, joint overload is not the only risk factor in this disease. For instance, the presence of OA in non-weight-bearing joints such as the hand suggests that metabolic factors may also contribute to its pathogenesis. Recently, white adipose tissue (WAT) has been recognized not only as an energy reservoir but also as an important secretory organ of adipokines. In this regard, adipokines have been closely associated with obesity and also play an important role in bone and cartilage homeostasis. Furthermore, drugs such as rosuvastatin or rosiglitazone have demonstrated chondroprotective and anti-inflammatory effects in cartilage explants from patients with OA. Thus, it seems that adipokines are important factors linking obesity, adiposity, and inflammation in OA. In this review, we are focused on establishing the physiological mechanisms of adipokines on cartilage homeostasis and evaluating their role in the pathophysiology of OA based on evidence derived from experimental research as well as from clinical-epidemiological studies.

## 1. Introduction

**Figure 1:**
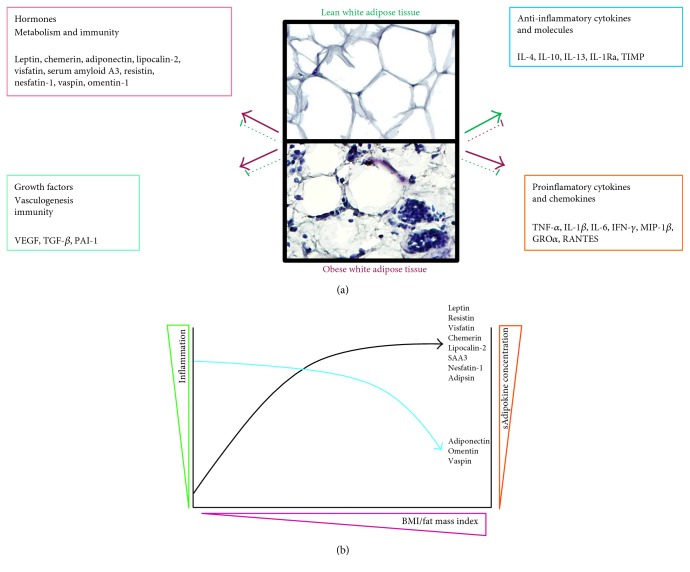
(a) Soluble mediators synthesized by white adipose tissue. Solid red arrows represent cytokines, growth factors, and hormones produced by obese white adipose tissue. Dotted red lines represent the inhibition of the soluble mediator expression by obese white adipose tissue. Solid green arrows depict endocrine and immune soluble mediators synthesized by lean white adipose tissue. Dotted green lines represent the inhibition of the soluble mediator expression by lean white adipose tissue. (b) Relationship of adipokines with the inflammation and the fat mass index in OA patients. sAdipokine: serum adipokine; BMI: body mass index.

Adipose tissue (AT) has emerged as a complex and highly dynamic organ with endocrine, metabolic, and immune regulatory roles. AT releases a plethora of bioactive peptides or proteins, immune molecules, and inflammatory mediators named “adipokines (only produced by the adipose tissue) or adipocytokines (primary but not exclusively produced by adipocytes)”. However, the term “adipokine” is used through the review to refer to these mediators (Figure 1(a)). Adipokines act both at autocrine/paracrine and at endocrine levels. To date, about a hundred adipokines constituting the adipokinome have been described to be synthesized by hypertrophic adipocytes from white adipose tissue (WAT). At first, it was thought that the adipokines were only involved in metabolic processes. However, at present, it is well known that adipokines represent a new family of compounds that act as key players in the complex network of soluble mediators involved in the vascular homeostasis, metabolism, and immunity. Adipokines are also involved in the pathophysiology of numerous diseases, including not only metabolic syndrome (insulin resistance, hyperglycemia, dyslipidemia, hypertension, and prothrombotic and proinflammatory states) but also rheumatic diseases such as rheumatoid arthritis, osteoarthritis (OA) and systemic lupus erythematosus, and cardiovascular and metabolic complications that are frequently observed in rheumatic diseases. Thus, adipokines exert potent modulatory actions on target tissues and cells involved in cartilage, synovium, bone, and various immune cells [1–6].

As a secretory organ, the AT has defining characteristics; it depends on fat depots (visceral or subcutaneous), the cell type composition (mature adipocytes, stromal-vascular cells, and nonfat cells including macrophages), and so on. In human obesity, AT is characterized by adipocyte hypertrophy and hyperplasia, macrophage infiltration, endothelial cell activation, and fibrosis. Adipocyte size is related to dysregulated adipokine and chemokine production; thus, the hypertrophic adipocytes modify their expression of proinflammatory mediators [4].

Mature adipocytes represent 50–85% of the total cellular components of WAT. Obese subjects are characterized by a slightly larger adipocyte number than that in lean individuals of which 10% is renewed annually. Intra-abdominal fat only represents 15% of the total fat in lean and obese individuals [7]. In obese individuals, AT from visceral fat is constituted of adipocytes, preadipocytes, fibroblasts, endothelial cells (stromal-vascular fraction), and bone marrow-derived activated macrophage human leukocyte antigen^+^ (HLA-DR^+^) infiltration as well as a small proportion of CD8^+^ T cells, natural killer T cells, mast cells, and B cells [8, 9]. Additionally, in obesity, there is a shift in the M2 (anti-inflammatory)/M1 (proinflammatory) balance, due to the migration of inflammatory monocytes from the periphery to macrophage cluster surrounding necrotic adipocytes. M1 are responsible for the circulating levels of inflammatory mediators, determining the chronic and systemic obesity-related inflammation [10].

Obesity not only has been a public health problem by enhancing the cardiovascular disease and metabolic disorders but also it has long been considered a risk factor for OA [11–13]. It has been reported that obesity increases the incidence of OA, particularly in weight-bearing joints such as the knees, and weight reduction is associated with a slower OA progression. A prevailing hypothesis is that obesity increases mechanical loading across the articular cartilage, leading to its eburnation, degradation, and degeneration [14]. However, obesity is also associated with OA in non-weight-bearing joints, such as those of the fingers, hands, and wrists, or temporomandibular joints which suggests that metabolic factors contribute to the high prevalence of OA in obese individuals [15]. All known adipokines are markedly dysregulated not only in obesity but also in type 2 diabetes or metabolic syndrome, where adipokines such as resistin, leptin, chemerin, and visfatin-1 implicated in the pathogenesis of inflammation and insulin resistance are overexpressed, and some adipokines with anti-inflammatory properties, such adiponectin and omentin, are decreased [16–21].

In this review, we are focused on establishing the physiological mechanisms of adipokines and their role in the pathophysiology of OA.

## 2. Adipokines and Their Receptors

### 2.1. Leptin

Leptin, from the Greek root *leptos*, meaning “thin”, was the first adipocyte-derived hormone/adipokine described. It is a nonglycosylated polypeptide of 146 amino acids and 16 KDa encoded by the gene obese (*ob*) in the chromosome 7q31.3 [22]. Leptin's three-dimensional structure is highly similar to the members of the long-chain helical cytokines, such as interleukin (IL)-6, IL-11, IL-12, and granulocyte colony-stimulating factor (G-CSF) [23–25]. The cytokine-like structure of leptin is indicative of its function in regulating immune responses. Leptin is mainly produced in WAT by mature adipocytes, but brown AT, intestine, placenta, mammary glands, gastric fundic epithelium, skeletal muscle, brain, joints (chondrocytes, synoviocytes, and infrapatellar fat pads [IFP]), and bone (osteophytes) also produce it. Its concentration fluctuates during the day, with its peak during the night, usually being higher in postpubertal women. The leptin production has shown a positive correlation with BMI and fat mass [26]. Leptin expression is also regulated by a wide range of inflammatory mediators such as lipopolysaccharide (LPS) and cytokines (tumor necrosis factor- (TNF-) *α*, IL-6, and IL-1*β*) during acute inflammatory responses [24, 27, 28].

It was first described as a satiety- and appetite-regulating hormone that induces a decrease of food intake, stimulating the hypothalamus inducing anorexigenic factors and suppressing orexigenic factors evoking the feeling of satiety, but also stimulates thermogenesis and energy expenditure by lowering blood glucose levels [29, 30]. The coexistence of an increasing of the body fat content and obesity in animal models and humans correlates with higher levels of leptin; this is widely interpreted as evidence of “leptin resistance”. It has been characterized by a decrease in transport of leptin across the blood–brain barrier and by elevated hypothalamic levels of SOCS3 and endoplasmic reticulum (ER) stress, which inhibit leptin signaling [31–37].

Leptin exerts its biological actions through its dimerized receptor, ObR [38, 39]. Six OB-R isoforms have been described, 4 short isoforms (Ob-Ra, Ob-Rc, Ob-Rd, and Ob-Rf), a soluble isoform Ob-Re, and the longest isoform (Ob-Rb) which is the only one with a full intracellular domain capable of transducing the leptin-binding signal [40]. Ob-Rb shows sequence homology to members of class I cytokine receptor (gp130) superfamily which includes the IL-6R, leukocyte inhibitory factor receptor (LIFR), and G-CSFR [38, 39]. The Ob-Rb is expressed in the brain, erythrocytes, blast cells, hematopoietic CD34^+^ stem cells, and various subpopulations of CD4^+^ and CD8^+^ T and B cells, dendritic cells, monocytes, neutrophils, macrophages, and natural killer cells (NKs) [39, 41, 42]. The Ob-Rb lacks intrinsic tyrosine kinase activity. Nonetheless, it has been shown to have the signaling capabilities of IL-6R (gp130), activating Janus kinases (JAK) 2, signal transducers, and activators of transcription (STAT) 3 signaling pathway. However, alternative pathways in immune cells have been described, such as extracellular signal activated kinase (ERK)1/2, p38, Jun N-terminal kinases (JNK), protein kinase C (PKC), Src-homology 2 domain-containing phosphatase 2 (SHP2)/growth factor receptor-bound protein 2 (GRB2), and phosphatidylinositol 3 kinase (PI3K)/K9/protein kinase B (AKT) pathways. This hormone circulates as an active free form and bound to plasma proteins and the soluble receptor isoform (Ob-Re) [8, 24, 41–50].

Regarding molecular mechanisms of attenuation of leptin signaling under conditions of continuous stimulation, it has been demonstrated that the two proximal intracellular tyrosine residues (Tyr985 or Tyr1077) in LEPRb were sufficient for the attenuation of STAT3 activation [51].

The central effects of leptin in innate immunity involve the activation of proliferation and phagocytosis of monocytes/macrophages, the chemotaxis of neutrophils, the release of oxygen radicals by these cells, and the activation of NK cells. Leptin also upregulates the secretion of proinflammatory cytokines (TNF-*α*, IL-6, and IL-12) by macrophages [25, 52, 53].

On adaptive immunity response, leptin strikingly stimulates the proliferation of naïve T cells and IL-2 production through mitogen-activated protein kinases (MAPK) and phosphatidylinositol 3 kinase (PI3K) pathways. Leptin has significant role in promoting polarization towards Th1 cell response. Studies in humans have demonstrated the role of leptin in the activation of lymphocytes. Leptin alone is unable to induce the proliferation and activation of mature circulating T lymphocytes unless it is coadministered with other nonspecific immunostimulants (PHA or Con A), in which case, leptin results in the induction of early (CD69) and late activation markers (CD25 and CD71) in both CD4 and CD8 lymphocytes [25, 41, 42, 54]. Moreover, it has been shown that Tregs produce leptin and express its receptor. Leptin acts as a negative signal in proliferation of Treg cell [55].

Circulating levels in normal lean individual are 5–15 ng/mL, whereas in subjects with obesity, these levels can reach 100 ng/mL and exceed 250 ng/mL in the morbidly obese [56].

### 2.2. Adiponectin

Adiponectin or adipocyte complement-related protein 30 kDa (Acpr30) is a protein of 244 amino acids produced by adipocytes, placenta, the liver, epithelial cells, osteoblasts, myocytes (in response to inflammatory stress or in response to metabolic and/or oxidative aggression), and by pituitary cells. In the blood stream, adiponectin is in three forms: trimer (low molecular weight), hexamer (medium molecular weight), and 12- to 18-mer (high molecular weight). Also, a globular adiponectin results from the cleavage of the full-length monomer [57–60], being the high molecular weight isoform and the most biologically active [61]. Adiponectin consists of 4 regions: a short signal sequence, a short region that varies between species, a 65-amino acid region similar to collagenous proteins, and a globular domain [61]. Circulating adiponectin levels are negatively correlated with the BMI and decreased in obese subjects, type 2 diabetes, and cardiovascular disease [19, 62, 63].

AdipoR1 and AdipoR2 are the major receptors for adiponectin. AdipoR1 is abundantly expressed in the muscle, hypothalamus, brainstem, and pituitary gland while AdipoR2 is expressed in the liver, astrocytes, and cortex. AdipoR1 is more tightly linked to the activation of AMPK, p38-MAPK, JNK, peroxisome proliferator-activated receptor- (PPAR-) *α*, and nuclear factor- (NF-) *k*B pathways that regulate the inhibition of gluconeogenesis together with increased fatty acid oxidation, while AdipoR2 is more involved in the activation of the PPAR-pathways, which stimulate energy dissipation by increasing fatty acid oxidation and inhibit oxidative stress and inflammation. T-cadherin has also been reported as a receptor for high molecular multimers of adiponectin [63–68]. Adiponectin may attenuate TNF-*α*, IL-6, MCP-1, vascular cell adhesion molecule-1 (VCAM-1), intercellular adhesion molecule-1 (ICAM-1), and endothelial-leukocyte adhesion molecule 1 (ELAM-1) expression, inflammation, oxidation, and fibrosis in AT through the inhibition of NF-*k*B activation [69–71]. Moreover, adiponectin suppresses superoxide radical generation in endothelial cells. Adiponectin acts by inhibiting proinflammatory response, polarizing macrophages from M1 to M2, and Th1/Th17 to Th2/Tregs, and inhibiting TLR4-mediated NF-*k*B activation [72, 73]. Circulating levels in normal lean individual are 11–15 *μ*g/mL, whereas with obesity, these levels can decrease 8 *μ*g/mL [19].

### 2.3. Resistin

Resistin or AT-specific secretory factor (ADSF) or C/EBP-epsilon-regulated myeloid-specific secreted cysteine-rich protein (XCP1) is a 12.5 kDa cysteine-rich adipose-derived peptide hormone, encoded by the *RETN* gene that belongs to the family of “resistin-like molecules” or “FIZZ” (found in inflammatory zone) [63, 74]. In mice, circulating resistin exists in a disulfide-linked hexamer or a smaller trimer. In humans, resistin is present in two quaternary forms: an abundant high molecular weight hexamer and a less abundant but more bioactive trimer, which induces hepatic insulin resistance and inflammation [17, 75].

The resistin expression in rodents is primarily by adipocytes, while in humans is mainly produced by monocytes and macrophages activated with LPS, IL-1*β*, IL-6, TNF-*α*, resistin itself, and in less extent by pancreatic *β* cell, lung cells, and placental tissue [63]. The relevance and physiological role of resistin in humans remain unclear. Given the incomplete homology (59%) between human and mouse resistin [74] and the absence in humans of one of the three murine resistin isoforms, resistin in humans may have a different physiological role than that in mice. Resistin appears to be a link between obesity and insulin resistance, and inflammation and insulin resistance in rodents. In humans, elevated circulating resistin levels are significantly related to increased risk of type 2 diabetes [17, 76], while resistin has been implicated in the pathogenesis of obesity-mediated insulin resistance and type 2 diabetes in rodent models [17, 63, 75, 77].

Resistin inhibits the anti-inflammatory effects of adiponectin on vascular endothelial cells by promoting the expression of the proinflammatory VCAM-1, ICAM-1, pentraxin 3, and proinflammatory cytokines (MCP-1, TNF-*α*, IL-6, and IL-12) through NF-*κ*B dependent pathway in these cells, thereby enhancing leukocyte adhesion and inflammatory process [78–80].

Resistin competes with lipopolysaccharide (LPS) for binding to TLR4 and adenylyl cyclase-associated protein 1 (CAP-1) [79, 81, 82]. Some other potential receptor candidates including an isoform of decorin involved in WAT expansion, tyrosine kinase-like orphan receptor-1 (ROR1) in 3T3-L1 cells, or insulin-like growth factor 1 receptor (IGF-1R) in fibroblasts from rheumatoid arthritis patients have also been described [83]. Thus, resistin could interact with different receptors depending on the tissue and cell types. Resistin activates G protein-dependent mechanism, the adenylate cyclase/cAMP/PK A pathway, the PI3-kinase/Akt pathway, the PKC, and extracellular Ca^2+^ signaling through L-type voltage-sensitive Ca^2+^ [3, 84–86].

### 2.4. Visfatin

Visfatin or pre-B cell colony-enhancing factor 1 (PBEF1) or nicotinamide phosphoribosyl transferase (NAmPRTase or Nampt) is a 52 kDa enzyme of 491 amino acids that promotes B cell maturation, stimulates the expression of proinflammatory cytokines and chemokines (IL-1*β*, IL-6, TNF-*α*, and SDF-1 or CXCL12), VEGF, and MMP-2/9, and inhibits neutrophil apoptosis [87, 88]. Visfatin was described to be a highly expressed protein with immune cell signaling and nicotinamide adenine dinucleotide (NAD) biosynthetic activity, which is essential for pancreatic *β* cell function; thus, visfatin presents an insulin-like effect [89]. Visfatin was predominantly found in visceral WAT, muscle, bone marrow, liver, lymphocytes, macrophages that infiltrate AT, and fetal membranes [63]. Possible correlations between circulating visfatin and anthropometric or metabolic parameters in obesity, overweight, type 2 diabetes, adiposity, metabolic syndrome, and cardiovascular disease have been determined in some studies [63, 90]. Visfatin's receptor is currently unknown. However, it is well known that there are 3 signaling pathways activated by visfatin: the first one is mediated by the *β*1 integrin and involves signaling through the ERK, p38 MAPK NF-*k*B, and AP-1 pathways; the second one is mediated by IL-6 and involves STAT3, Nampt, and Sirt-1 and Sirt-6; and the third one involves redox pathways and the reduction of reactive oxygen metabolites through increased activity of superoxide dismutase (SOD), catalase (CAT), and glutathione peroxidase (GSHPx) [88, 91–93].

### 2.5. Chemerin

Human chemerin also known as retinoic acid receptor responder protein 2 (RARRES2) or tazarotene-induced gene 2 protein (TIG2) is composed of 163 residues and a molecular weight of 16 kDa [94]. Chemerin is expressed as a precursor (prochemerin) that is cleaved at the C-terminus by a serin protease to become active [95]. It is expressed in the spleen, lymph nodes, and lung. Adipocytes, perivascular AT stroma-vascular cells, and vascular smooth muscle cells secrete physiological amounts of chemerin in early adipocyte differentiation, and when adipocytes are mature, chemerin production is increased. It is an attractant for immune cells and may play a role in the recruitment of tissue macrophages, and it has been identified as an adipokine of the metabolic syndrome [96]. Chemerin is upregulated in WAT cells upon IL-1*β* stimulation in vitro, and chemerin serum levels are increased in obese patients; thus, chemerin may be the functional link between chronic low grade inflammation, obesity, type 2 diabetes, and cardiovascular diseases. Chemerin exerts its functions by binding to the G protein-coupled receptor ChemR23 or CMKLR1 (chemokine-like receptor 1), GPR1, and CCRL2 (chemokine C-C motif receptor) [97–100]. CMKLR1 is expressed by activated monocytes/macrophages, NKs, and foam cells, while GPR1 is expressed in the liver, intestine, kidney, and AT. CCRL2 is produced by lung endothelial cells and liver endothelium. ChemR23 is also expressed by endothelial cells, where it is upregulated by proinflammatory cytokines, and strongly induces angiogenesis in vitro by promoting endothelial cell proliferation through VEGF and adhesion molecule expression (ICAM and E-selectin) and remodeling by the stimulation of gelatinolytic matrix metalloproteinase (MMP) activity (MMP-2, MMP-9) [101–103]. Positive correlations were detected between chemerin serum levels and BMI, fasting insulin, leptin, and CRP [104].

### 2.6. Lipocalin 2

Lipocalin 2 (LCN2), also known as neutrophil gelatinase-associated lipocalin (NGAL), siderocalin, 24p3, or uterocalin, belongs to the lipocalin protein superfamily [105, 106]. LCN2 is a 25 kDa glycoprotein and binds and transports various small lipophilic substances such as retinoids, arachidonic acid (Leukotriene B4), and steroids. LCN2 protein is present as a 25 kDa monomer, as a 46 kDa homodimer, and in a covalent complex with MMP-9. LCN2 a mammalian acute-phase protein also involved in iron homeostasis (ferritin and transferrin-independent iron delivery) is highly expressed in response to toxic amyloid *β*_1–42_ peptides and which is related to cell proliferation and apoptosis of hematopoietic cells [107].

Lipocalin 2 is abundantly expressed in WAT and is induced by inflammatory stimuli through activation of NF-*k*B. Serum concentrations of this protein are positively associated with adiposity, hyperglycemia, insulin resistance, and CRP levels. LCN2 binds at least two mammalian surface receptors, LCN2 receptor (also known as 24p3R, NGALR, or SLC22A17), a brain-type organic cation transporter (BOCT), and megalin (also known as low-density lipoprotein receptor-related protein 2, LRP2, gp330), a multiligand scavenger receptor [108, 109].

### 2.7. Vaspin

Vaspin, a visceral AT-derived serpin (serpinA12) is known mainly for its insulin-sensitizing effects and modulatory role on glucose tolerance. This 50 kDa adipokine was first discovered in a rat model when identifying genes that were differentially expressed during the development of obesity and type 2 diabetes [110]; vaspin level is low in obesity and suppresses leptin, TNF-*α*, ICAM, and resistin synthesis [111, 112]. Subsequently, decreased vaspin levels have been reported to be linked to diabetes, metabolic syndrome, obesity, coronary artery disease, and impaired insulin sensitivity [113]. Vaspin interacts with GRP78, a cell membrane glucose-regulated protein, to induce intracellular signaling in vascular smooth muscle cells that inhibits reactive oxidative species, MAPK, PI3K/Akt, and the phosphorylation of NF-*k*B and PKC*θ* induced by TNF-*α*, which improves glucose and lipid metabolism and relieves metabolic dysfunction and inflammatory responses in obesity [111, 113, 114].

### 2.8. Omentin

Omentin is a secretory protein of 34 kDa; it is highly and selectively expressed in visceral stromal-vascular cells compared with subcutaneous AT. Other tissues and cells (small intestine Paneth cells and endothelial cells) also express omentin-1 or intelectin, intestinal lactoferrin receptor, or endothelial lectin at lower level bacteria [115]. Omentin, as adiponectin, may play a paracrine or endocrine role in modulating insulin sensitivity and as type of Ca^2+^-dependent lectin with affinity for galactofuranosyl residues, in gut immunity against pathogenic bacteria or their components [21, 116].

Omentin plays a role in inflammatory response favoring downregulation of inflammation and cell differentiation by AMP/eNOS signaling pathway [21, 116]. Circulating levels are inversely correlated to obesity and BMI, waist circumference, and leptin in healthy subjects; the normal level is reported in 0.37 *μ*g/mL, but are significantly reduced in Crohn's disease, synovial fluid of rheumatoid arthritis patients, other inflammatory diseases, and obese individuals to 0.31 *μ*g/mL [116]. A definitive omentin receptor has not yet been identified. Nonetheless, omentin signaling pathway involves AMPK and eNOS and inhibits Akt pathways, C-reactive protein (CRP) production, TNF-*α*, adhesion molecules, TLR4, and NF-*k*B signaling pathways [117].

### 2.9. Nesfatin-1

Derived from the protein nucleobindin 2 (NUCB2), nesfatin-1 is identified in 2006 as an anorexigenic peptide of 82 amino acid lengths that regulates appetite and body weight [118]. Nesfatin-1/*NUCB2* is expressed in hypothalamic nuclei, the arcuate nuclei, lateral hypothalamus, paraventricular nuclei, supraoptic nuclei, gastric mucosa, pancreatic islets, testis, and AT [119]. Nesfatin-1 secretion from AT, particularly subcutaneous adipose depots, is negatively correlated with BMI, body weight, percentage body fat, body fat weight, and fasting blood glucose and is increased by proinflammatory cytokines (IL-1*β*, TNF-*α*, and IL-6) as well as insulin and dexamethasone [120]. In obesity, nesfatin-1 is suggested to play a role in the enhancement of lipid accumulation pathways [121]. Nesfatin activates G protein-dependent mechanism and extracellular Ca^2+^ signaling through L-, P/Q-, or M-type voltage-sensitive Ca^2+^ channels [122–124].

## 3. Adipokines in the Pathobiology of Osteoarthritic Cartilage

Chronic inflammatory disease results from a failure or absence of the mechanisms responsible for maintaining homeostasis and the persistence of the mechanisms that upregulate inflammation. Adipokines in the joint cavity from OA patients are produced majorly by IFPs and synoviocytes, but chondrocytes, inflammatory cells, and osteoblasts as well as osteoclasts release also large amounts of adipokines inducing and perpetuating the inflammatory state [125, 126].

### 3.1. Leptin

Leptin and Ob-Rb have been isolated from chondrocytes, synoviocytes, osteophytes, and IFPs from patients with OA [127].

A seminal study showed that leptin deficient (*ob/ob*) or leptin receptor deficient (*db/db*) female C57BL76J mice had a tenfold increase in adiposity and morbid obesity compared with controls. However, it was not associated with the increased incidence of knee OA. Systemic inflammatory cytokine levels remained without change, and the subchondral bone morphology was unaltered suggesting that obesity alone was unable to induce knee OA. The results pointed a preponderant role for leptin in the development of OA by regulating both the skeletal and immunological response [128].

On the other hand, in vivo leptin injection into the rat knee joints showed cartilage degradation by increasing MMP and cysteine proteases in vitro, it has been demonstrated that OA chondrocytes produce more leptin than normal chondrocytes, and it stimulates chondrocytes to secrete TNF-*α*, IL-1*β*, IL-6, IL-8, growth-related oncogene (GRO), and MCP-1 and reduces proliferation of OA chondrocytes [129].

**Figure 2:**
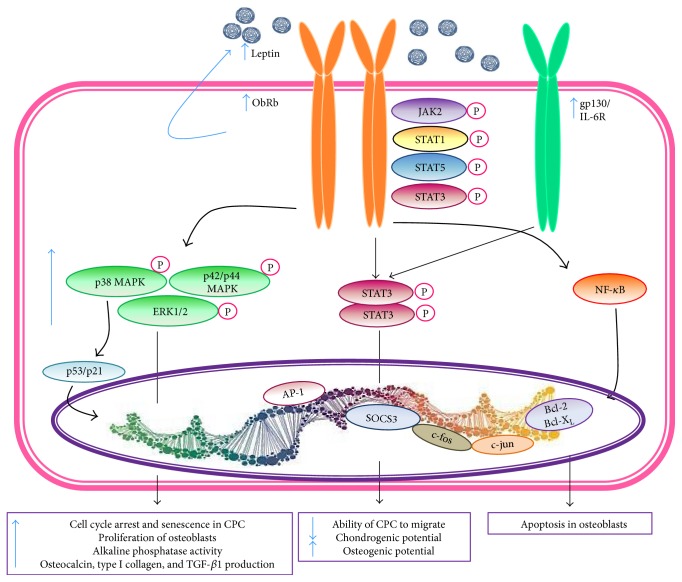
Leptin signal transduction. The Ob receptor b (ObR b) isoform of leptin binds to the JAK-STAT intracellular signaling system. As a consequence of leptin binding to its receptor, JAK2 is activated by the autophosphorylation. STAT1 and STAT5 bind tyrosine residues. STAT3 proteins form dimers and translocate to the nucleus and regulate *c-fos*, *c-jun*, SOCS3, and AP1 gene expression. Src homology domains of receptor (SHP2) activate MAPK pathways (p38, p42/44, and ERK1/2). These pathways induce the expression of cytokine and chemokine genes. Moreover, ObRb/leptin also induces the transcription of metalloproteinases and aggrecanases, cartilage degradation proteins, and the signaling pathways of inflammatory cytokines through activation of NF-*k*B and AP-1 that transcribe the genes of inflammatory proteins (IL-1*β*, IL-6, TNF-*α*, and induced nitric oxide synthase among others). Leptin, through interleukin 6 (IL-6)/gp130 pathway activates STAT3, which in the nucleus, transcribes the gene of SOCS3 that suppresses the leptin signaling pathways. Bcl: B cell lymphoma; ERK: extracellular signal-regulated kinase; JAK: c-Jun N-terminal kinase-associated kinase; MAPK: mitogen-activated protein kinase; NF-*k*B: nuclear factor-kappa B; ObR: Ob receptor; SOCS3: suppressor of cytokine signaling-3; STAT: signal transducer and activator of transcription.

Regarding the signaling leptin pathways in OA, it involves MAPKs (p38, JNK, and MEK) and NF-*k*B as it has been demonstrated in chondrocyte cultures treated with recombinant human leptin. Leptin induces degradation of aggrecan by upregulating disintegrin and metalloproteinase with thrombospondin motif- (ADAMTS-) 4, 5, and 9 and MMP-1, MMP-2, MMP-3, MMP-9, and MMP-13 [130–132]. Leptin has also been involved in the expression of proinflammatory cytokines by synoviocytes (IL-6, IL-8), chondrocytes (IL-1*β*), cartilage explants (IL-6, IL-8, PGE_2_) via activation of NF-*κ*B, VCAM-1 expression by chondrocytes via JAK2 and PI3K signaling pathway, and chondrocyte apoptosis, phenotype loss, and decrease of proliferation. (Figure 2 and Table 1) [133].

Leptin also synergizes with IL-1*β*, a classic proinflammatory cytokine involved in cartilage damage enhancing the production of iNOS, prostaglandin E_2_ (PGE_2_), and cyclooxygenase-2 (COX-2) in human chondrocytes [28].

In OA, leptin has been demonstrated to act as a proinflammatory agent by decreasing in the ability of the chondrogenic progenitor cells to migrate, inducing the production of proinflammatory cytokines and matrix metalloproteinase- (MMP-) 1, 2, 3, 9, and 13 expression and the chondrocyte senescence by activating the p53/p21 pathway as well as nitric oxide (NO) (Figure 2 and Table 1). NO promotes apoptosis, chondrocyte phenotype loss, and MMPs activation [134].

There is also a difference in the expression of the leptin receptor (Ob-Rb) between the lateral and medial compartment, with a larger expression of the receptor in the latter, and this could be associated with the asymmetrical damage in this disease [134].

Nonetheless, chondrocytes and synoviocytes are not the sole targets of leptin; osteoblasts are also a significant target of leptin action. OA subchondral osteoblasts produce twofold leptin and Ob-Rb than the normal cells, and this abnormal production by OA osteoblasts could be responsible, in part, for the osteoblasts differentiation and proliferation and the elevated levels of alkaline phosphatase activity, osteocalcin release, type I collagen, and TGF-*β*1 production (metabolic markers in osteoblasts), since the leptin inhibition assays with piceatannol and tyrphostin (selective inhibitors of JAK2/STAT3 and JAK1/STAT3, resp.) or with the use of inactivating antibodies against leptin or the use of small interfering RNA (siRNA) reduced the expression of MMP-13, metabolic markers in osteoblasts, and endochondral ossification as well as bone mineralization [135].

It has been demonstrated that serum and plasma leptin levels in knee OA patients correlate positively with BMI, more specifically with the fat mass and central adiposity (Figure 1(b)) [136–138]. Also, a meta-analysis that included 11 case-control clinical studies and 3625 subjects showed that plasma leptin concentrations were higher in OA patients compared with controls and higher in premenopausal women than in men [139].

**Figure 3:**
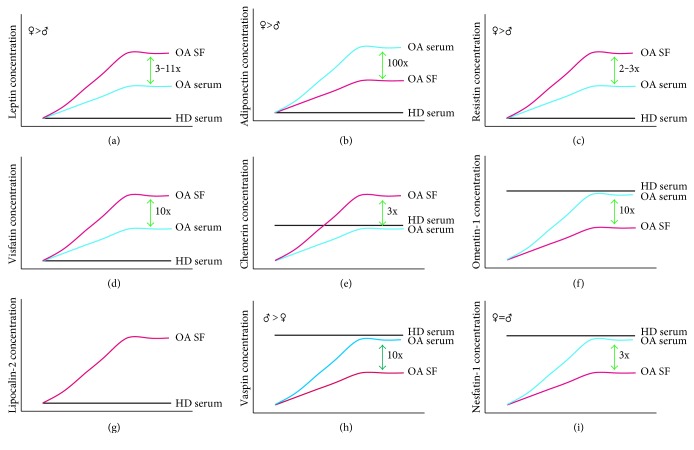
Adipokines in synovial fluid and serum from OA patients and their relative expression compared with healthy individuals. (a) Leptin levels, (b) adiponectin levels, (c) resistin levels, (d) visfatin levels, (e) chemerin levels, (f) omentin-1 levels, (g) lipocalin-2 levels, (h) vaspin levels, and (i) nesfatin-1 levels. Red lines: synovial fluid concentration in patients with OA; blue lines: serum concentration in patients with OA; black lines: serum concentrations in healthy donors; SF: synovial fluid; HD: healthy donors; ♀: female levels; ♂: male levels.

Leptin and its soluble receptor (sObR) have been detected in the synovial fluid (SF) obtained from OA patients, and interestingly, leptin levels measured in the joint fluid exceed three- to elevenfold than those found in serum (Figure 3(a)) [140]. In addition, Ku et al. and Karvonen-Gutierrez et al. reported that SF and serum leptin levels are directly correlated with the radiographic severity of OA [141, 142] and also with proinflammatory cytokines, MMP-1, and MMP-3 levels in OA patients, suggesting the possible use of leptin as a potential biomarker for quantitative detection of OA severity. In a recent study, SF leptin concentrations were associated also with knee and hip pain in OA patients [140, 143, 144]. In addition, in Chinese population, serum levels of leptin were independently associated with increased knee cartilage volume assessed by radiography [145]. Moreover, leptin and the sObR were also highly correlated with greater cartilage volume loss using high-resolution 3D MR images [146, 147].

Based on these studies, upregulated expression levels of leptin may be a risk factor in OA and it could be used as a very sensitive biomarker for predicting the severity of the disease, pain, and cartilage damage [139, 148].

### 3.2. Adiponectin

**Figure 4:**
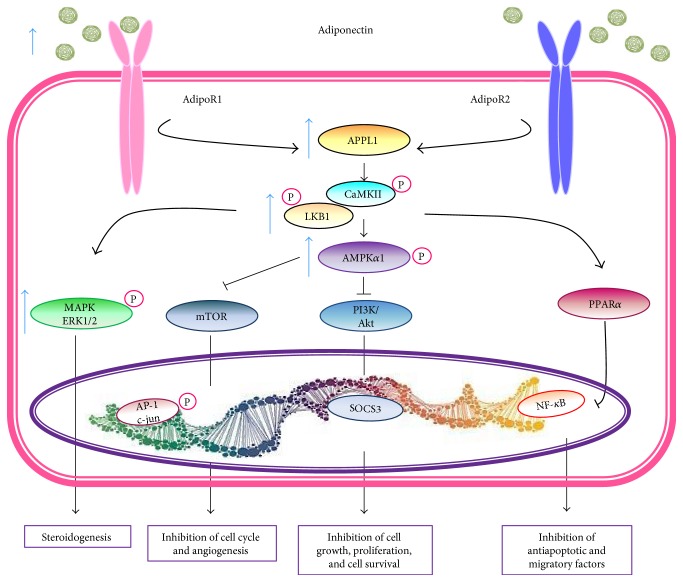
Adiponectin signaling via AdipoR1 and AdipoR2 activation. Adiponectin is decreased in obesity. AdipoRs can lead to stimulation of various signaling pathways. AMPK blocks angiogenesis via mTOR and cell growth and proliferation via PI3K/Akt. Antiapoptotic and migratory proteins induced by p65/p50 of the NF-*k*B pathway is inhibited by PPAR-*α*. Adipo R: adiponectin receptors; APPL1: adaptor protein containing pleckstrin homology domain, phosphotyrosine-binding domain, and leucine zipper motif 1; PPAR-*α*: peroxisome proliferator-activated receptor *α*; AMPK: 5′-adenosine monophosphate-activated protein kinase; MAPK: mitogen-activated protein kinase; ERK1/2: extracellular signal-regulated kinases 1/2; SOCS3: suppressor of cytokine signaling-3; mTOR: mammalian target of rapamycin; LKB1: liver kinase B1.

Adiponectin has been implicated in OA pathogenesis on the basis of both clinical and experimental observations. In cultured chondrocytes, full-length adiponectin at physiological and high concentration (5–30 *μ*g/mL) is able to induce several proinflammatory molecules and mediators, such as NOS2, IL-6, IL-8, vascular endothelial growth factor (VEGF), MCP-1, CC-chemokine ligand 2 (CCL-2), VCAM-1, ICAM-1, and MMPs (1, 3, 2, 9, and 13) [129, 149–151]. Moreover, elevated levels of AdipoR1 and in lesser extent of Adipo R2 are expressed by OA cartilage and they have been associated with catabolic effects of adiponectin [152, 153]. Interestingly, some studies have shown a protective role of adiponectin in knee OA. Cultured OA chondrocytes pretreated with full-length adiponectin at low concentration (less than 1 *μ*g/mL; as it has been determined in SF from OA patients) downregulated MMP-13 and PGE_2_ induced by IL-1*β* and upregulated tissue inhibitor of metalloproteinase- (TIMP-) 1 and 2 expression [150, 154]. In human knee OA synovial fibroblasts (OASFs), adiponectin was found to induce expression of ICAM-1 via the liver kinase (LK) B1/calmodulin-dependent protein kinase II (CaMKII), AMPK, c-Jun, and AP-1 signaling pathway and this expression increased the adhesion of monocytes to human OASFs (Figure 4 and Table 1) [151].

By contrast, there are many reports that have demonstrated that physiological concentrations of adiponectin induce the release of anti-inflammatory mediators including IL-10, IL-1Ra, TIMP-1, and TIMP-2 by human chondrocytes and macrophages (Figure 4 and Table 1) [154–156].

Furthermore, adiponectin acts as a modulator of macrophage phenotypes. It switches the phenotype from the proinflammatory classically activated macrophage (M1) to an anti-inflammatory alternatively activated macrophage (M2) [73]. Obese adipose tissue is predominantly enriched with M1 polarized macrophages, which causes exacerbation of inflammation and tissue destruction, while M2 macrophages exert an anti-inflammatory action and protect against obesity-related metabolic disorders. Adiponectin knockout mice display increased expression levels of M1-related genes, such as TNF-*α*, IL-6, and MCP-1, in peritoneal macrophages and stromal-vascular fractions compared to wild-type mice [73]. Treatment of wild-type mice with adiponectin stimulates the expression of M2-related genes, including arginase-1, IL-4, IL-10, and macrophage galactose N-acetyl-galactosamine specific lectin-1 [73]. Adiponectin also promotes the polarization of human monocyte-derived macrophages into anti-inflammatory M2 macrophages through a PPAR-*α*- and AMP-activated protein kinase-dependent mechanism [157]. It has been shown that adiponectin polarizes Kupffer cells and RAW264.7 macrophages to M2 through a mechanism involving the AdipoR2 via IL-4/STAT6- and MyD88-dependent mechanism [158]. Additionally, adiponectin bound to calreticulin/CD91 promotes and enhances the ability of macrophage to remove early opsonized apoptotic cells, which is crucial in preventing exacerbated inflammation and immune system dysfunction [159]. Finally, the globular form of adiponectin has a high-binding affinity for the receptor AdipoR1. Elevated levels of AdipoR1 have been associated with the gene expression of type II collagen, aggrecan, and sex determining region-box 9 (SOX9) which suggest a role of adiponectin in cartilage repair and remodeling [150]. Hence, these data suggest that adiponectin induces anti-inflammatory profile and reduces chronic inflammation in target organs thereby leading to protection against various obesity-related disorders.

The importance of adiponectin in the pathogenesis of OA is also supported by clinical observations. Plasma adiponectin levels are negatively correlated with BMI (Figure 1(b)) [137]. Adiponectin levels are significantly lower in patients with OA than in healthy controls, and knee OA patients with higher radiographic severity had significantly lower adiponectin levels in both plasma and SF [145, 160]. In addition, adiponectin levels detected in OA synovial fluid were almost 100 times lower than those in plasma and it correlated with OA severity and aggrecan degradation but not with type II collagen (Figure 3(b)) [154, 160]. Remarkably, the adiponectin : leptin ratio in the synovial fluid has been proposed as a predictor of pain in knee OA [161]. A lower leptin : adiponectin ratio correlated with lower knee OA pain [143]. Furthermore, it has determined that weight loss is associated with an increase in adiponectin and a decrease in leptin and cartilage oligomeric matrix protein plasma levels in obese subjects [144]. This suggests that lower levels of adiponectin are related to a more aggressive disease and that higher levels have anti-inflammatory properties.

Recently, serum adiponectin level was not associated with cartilage volume measurements by X-rays [145], whereas higher values correlated with less cartilage volume loss in the medial compartment of the femur when evaluated by high-resolution 3D MR image [147]. Furthermore, also the serum level was positively associated with infrapatellar fat pad volume evaluated by MRI in OA patients after adjustment for age, sex, weight, and height, although this association became nonsignificant after adjustment for IL-17 [162].

On the other hand, plasma adiponectin levels were higher in women with erosive hand OA compared to those with nonerosive OA [163, 164]. Conversely, a study showed that patients with high adiponectin levels had a decrease risk for hand OA progression [165]. However, another study indicated no association between plasma adiponectin levels and radiographic hand OA severity [166].

Regarding OA of the shoulder, few studies showed a correlation between the levels of adipokines and pain, especially leptin, adiponectin, and BMI [167]. Nonetheless, the levels of leptin and adiponectin does not correlate with the functional limitation [168]. The presence of Ob-Rb, AdipoR1, and AdipoR2 on the shoulders from patients with late-stage OA has been reported [167]. The profile of adipokines in OA shoulder is different when compared with knee or hip OA, with a higher serum/SF ratio for leptin and adiponectin compared with knee OA as well as higher levels of adiponectin and lower levels of leptin in both serum and SF [169].

### 3.3. Resistin

**Figure 5:**
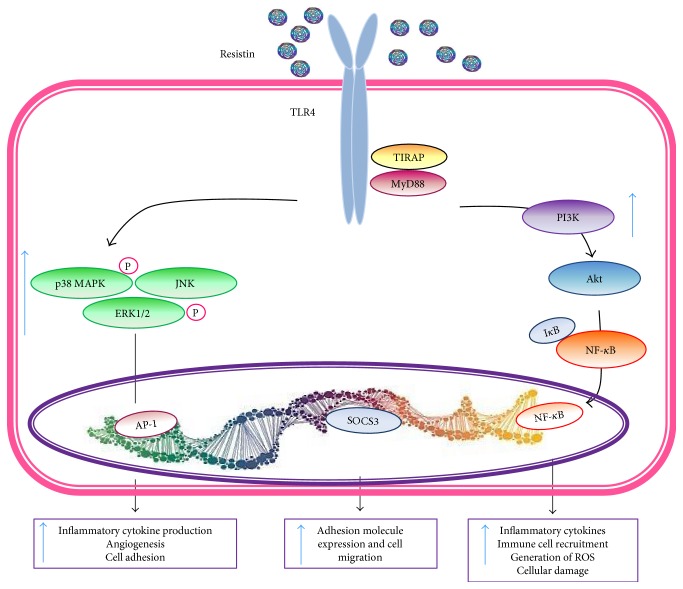
Resistin signaling. Resistin is recognized by TLR4 receptor. Two signaling pathways are triggered through the recruitment of the adaptor molecules TIRAP and MyD88. The first through PI3K followed by Akt and NF-*k*B. The second through MAPK pathway, followed by upregulation of NF-*k*B.

Resistin is produced mainly by articular WAT and in lesser extent by OA synovial tissue and osteophytes (osteoblasts and osteoclasts) and participates in adipogenesis, insulin resistance, meniscal GAG degradation, and inflammatory processes. It has a positive correlation with obesity, insulin resistance, and chronic inflammation [153, 170] (Figures 1(a) and 1(b)). In murine models, cartilage and in human primary chondrocytes resistin was also found to induce MMP13, ADAMTS4, PGE_2_, TNF-*α*, and IL-8 (Figure 5 and Table 1) [171]. Fascinatingly, leptin-deficient mouse models (*ob/ob* or *db/db*) have elevated concentrations of serum resistin, suggesting that resistin levels depend on leptin levels [172].

Resistin was found in plasma and SF of patients with OA. Circulating levels of resistin positively correlates with leptin levels and IL-6, MMP1, and MMP3 levels in SF, with no significant difference for diabetic versus nondiabetic patients or gender or hand OA [169, 173]. Resistin levels in females are significantly higher than those in males (Figure 3(c)).

In patients with radiographic changes of hand OA patients, plasma resistin levels were higher than in nonradiographic hand OA and controls [173]. In contrast in knee OA, resistin has neither been associated with cartilage volume assessed by radiography [145] nor by high-resolution 3D MR image [147]. However, among patients with knee OA and join effusion, its presence in SF is clearly associated with the Lequesne index, a validated questionnaire for pain and disability. This association persisted even controlling by anthropometric measurements and metabolic factors [174]. Similarly, another study also found an association with the WOMAC score and CTX-II and resistin level in synovial fluid of patients undergoing arthroscopic lavage [175]. Recently, among knee OA patients, serum level of resistin was significantly associated with Kellgren-Lawrence grading scores, WOMAC pain scores, physical functional scores, WOMAC total scores, and CTX-II [175].

In addition, some studies have shown that the menisci are more susceptible to inflammation produced majorly by resistin followed by leptin and adiponectin. This response was similar to the one induced by IL-1*β* [170].

### 3.4. Visfatin

**Figure 6:**
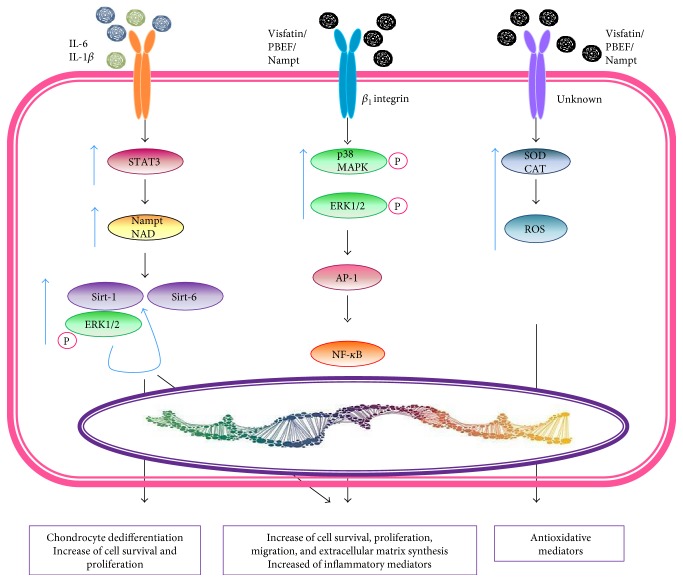
Visfatin signaling. Visfatin stimulates monocytes to release IL-6. IL-6 signals increase the expression level of STAT3 which upregulates the active enzymatic form of visfatin/PBEF/Nampt. Visfatin/PBEF/Nampt can increase cell survival through Sirt-1 and Sirt-6 stimulating the release of TNF-*α* inducing a chronic low grade inflammation. In the second pathway, visfatin signals through the cells surface receptor *β*1 integrin. This binding upregulates and activates p38 MAPK and ERK1/2. The MAPK cascade increases the expression of AP-1 and NF-*k*B that upregulate SDF-1, leading to increased survival and migration. The third pathway was demonstrated through the activation of unknown receptor increasing the antioxidative enzymes superoxide dismutase (SOD) and catalase (CAT).

Visfatin modulates the expression of chondrocyte extracellular matrix proteins. Human chondrocytes pretreated with visfatin inhibited IGF-1-stimulated proteoglycan synthesis in a dose-dependent manner by activating the extracellular signal-regulated kinases (ERK)/MAPK signaling pathway (Figure 6). Human OA chondrocytes produce visfatin, and IL-1*β*, IL-6, TNF-*α*, and glucocorticoids treatment increases visfatin synthesis [176, 177]. Moreover, IL-1*β* and IL-6 act synergistically with visfatin to increase the release of PGE_2_, MMPs, and NO, which is selectively blocked by small interfering RNA knockout of visfatin. Visfatin also induced ADAMTS4 and ADAMTS5 expression and MMP-3 and MMP-13 synthesis and release; it also reduced the synthesis of high molecular weight proteoglycans by immature mouse articular chondrocytes [176–179]. Moreover, high concentrations of visfatin decrease the expression of factors essential for the maintenance of chondrocyte phenotype including SOX9 and type II collagen [180, 181]. Taken together, all these data indicate that visfatin has a catabolic function in cartilage and might have an important role in the pathophysiology of OA (Table 1).

Besides, visfatin is expressed by OA mouse and human IFP in higher concentrations than in subcutaneous adipose tissue, especially next to the sites of osteophytes formation, also by synovial tissue, chondrocytes in osteophytes, osteoblasts, and osteoclasts in OA [126, 153, 182, 183]. The higher expression of visfatin in sites of high bone remodeling, combined with a reduced osteoclast differentiation and osteoclast specific markers, suggests a role in proinflammatory OA pathogenesis (Table 1) [153, 182]. OA patients have higher levels of circulating and local visfatin compared with controls, with higher amounts in SF versus matched plasma and more expression in OA IFP than in the matched subcutaneous AT (Figure 3(d)) [184]. Visfatin plasma and SF levels appeared to be positively associated with lipid metabolism, inflammation, C-reactive protein (CRP) levels, C-telopeptide of type II collagen (CTX-II), degradation biomarker of aggrecan, aggrecanases (AGG1 and AGG2), radiographic damage, and disease activity (Figure 1(b)) [172, 185]. It has been demonstrated that visfatin and IL-1*β* stimulate in a dose-dependent manner; the expression and release of nerve growth factor (NGF) by OA chondrocytes and NGF levels are involved in pain associated with knee OA [186], while hip OA pain has been associated with IL-6 and visfatin [140].

### 3.5. Chemerin

**Figure 7:**
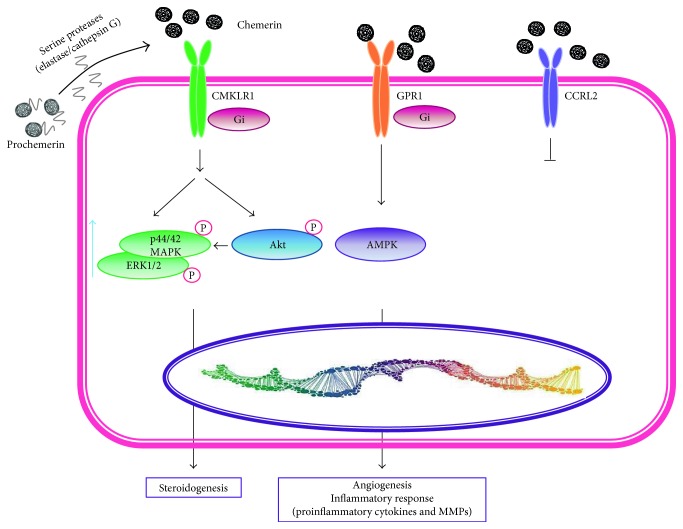
Chemerin signaling. Chemerin binds to three different G protein-coupled receptors: CMKLR1 (chemokine-like receptor 1), GPR1 (G protein-coupled receptor 1), and CCRL2 (chemokine (CC motif) receptor-like 2). The latter does not transduce any signal; once activated, CMKLR1 and GPR1 stimulate or inhibit different signaling pathways including MAPK ERK1/2, Akt, and AMPK to regulate different biological processes such as angiogenesis, inflammation, and steroidogenesis.

Chemerin (TIG2 or RARRES2) is a novel chemoattractant adipokine which directs leukocytes expressing CKMLR1, a G protein-coupled receptor, towards sites of inflammation (Figure 7).

Interestingly, human articular chondrocytes and resident cell in native cartilage express chemerin and its receptor [187]. Dexamethasone and IL-1*β* increases chemerin expression [188]. Furthermore, it has been demonstrated that recombinant chemerin enhances the production of several proinflammatory/procatabolic cytokines (IL-1*β*, IL-6, IL-8, and TNF-*α*) as well as MMP-1, MMP-2, MMP-3, MMP-8, and MMP-13 in human articular chondrocytes. Chemerin also induces angiogenesis in vitro by promoting endothelial cell proliferation, migration, and capillary tube formation. All of these elements play a key role in the turnover, degradation, and damage of the extracellular matrix, resulting in irreversible devastation of the cartilage in OA. Chemerin phosphorylates p42/44, MAPKs (ERK1/2) and Akt (Ser 473), both of which are involved in signal-transduction pathways that converge in inflammatory signaling (Figure 7) [187, 189].

Chemerin was detected in serum and SF from knee OA patients, and the serum concentration of this adipokine correlated with the disease severity in OA, BMI, and hsCRP (Figures 1(b) and 3(e)). However, no significant association was determined between serum chemerin concentration and age nor gender [190–192]. In addition, it has demonstrated that synovial tissue from knee OA patients express chemerin and its levels were also positively correlated with the severity of knee OA [192].

### 3.6. Omentin-1

**Figure 8:**
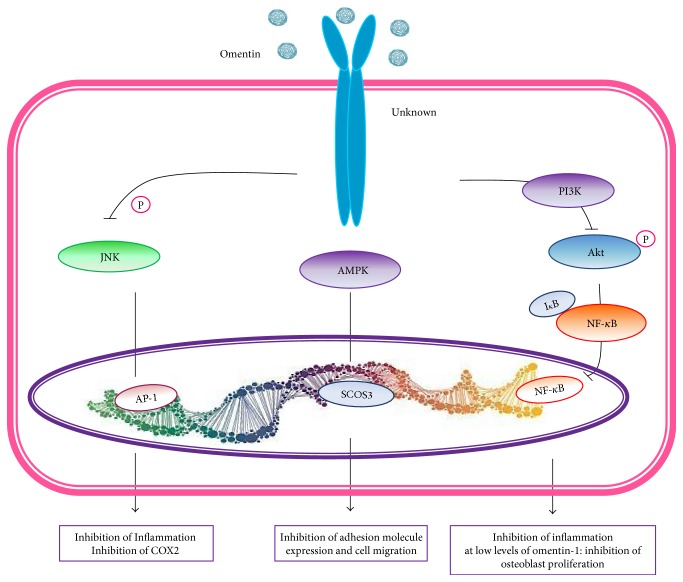
Omentin signaling. Omentin activates AMPK, which further blocks E-selectin expression and reduces endothelial inflammation. AMPK also activates endothelial nitric oxide (eNOS), also known as nitric oxide synthase 3 (NOS3) or constitutive NOS (cNOS), which has vasodilation effect and blocks JNK signaling. JNK activates inflammation through TNF-*α*-mediated COX2 effect. Moreover, omentin inhibits NF-*κ*B signaling pathway and thus inhibits inflammation.

Omentin-1 is a secretory protein that has also been identified as a new adipokine that is highly and selectively expressed in visceral AT. A recent study demonstrated that omentin-1 has a key role in the regulation of inflammation. The anti-inflammatory role of omentin has been supported by the findings that it prevents TNF-*α*-induced COX-2 inflammatory signal transduction through phosphorylation of AMPK/endothelial nitric oxide synthase (eNOS)/NO pathways. Moreover, omentin significantly inhibited the phosphorylation of JNK. Omentin plays an anti-inflammatory role by preventing the TNF-*α*-induced COX-2 expression in vascular endothelial cells. Besides, omentin-1 has been shown to reduce systemic release of inflammatory factors such as IL-6 [117]. Finally, omentin-1 has demonstrated to induce human osteoblast proliferation via the PI3K/Akt signaling pathway (Figure 8) [193].

Serum omentin-1 levels were not significantly different between the knee OA patients and healthy controls. Nevertheless, omentin-1 concentrations in SF were decreased significantly as the radiographic severity of OA was increased (Figure 3(f)). Moreover, SF omentin-1 levels were independently and negatively correlated with self-reported pain, radiographic severity, and physical disability in knee OA patients. Omentin-1 in SF might serve as a potential biomarker for reflecting the degenerative process and symptomatic severity of knee OA [194, 195]. Thus, it suggested that omentin-1 seems to have an anti-inflammatory role (Figure 1(b)).

### 3.7. Lipocalin 2

LCN2, a mechanoresponsive adipokine, has been identified in human chondrocytes, where IL-1*β*, TNF-*α*, IL-17, leptin, adiponectin, LPS, and dexamethasone are the major upregulators of its expression, while TGF-*β*1 and IGF-1 are the main downregulators. LCN2 exerts its effects through the receptors lcn2R/24p3R and megalin (gp330). LCN2 is likely to be involved in matrix degradation, as it forms molecular complexes with MMP-9 or gelatinase B [196–199]. LCN2 is expressed in both proliferating and hypertrophic growth plate zones of cartilage, and it induces type X collagen synthesis and decrease chondrocyte differentiation and proliferation [197]. LCN2 is induced in osteoblasts in the absence of mechanical loading, and it reduces osteoblast viability in the presence of iron and enhances the activity of MMP-9 released by osteoblasts. Furthermore, prestimulated human osteoblasts induce in a paracrine manner, LCN2 expression in human chondrocytes [198]. LCN2 promotes cartilage breakdown by blocking MMP-9 auto-degradation and by increasing chondroptosis [197, 200]. However, LCN2 appears to be not enough or necessary for OA cartilage destruction in mice [199].

Gupta et al. and Katano et al. confirmed that the level of LCN2 in SF was significantly higher in patients with RA than in those with OA (Figure 3(g)) [196, 200].

### 3.8. Vaspin

**Figure 9:**
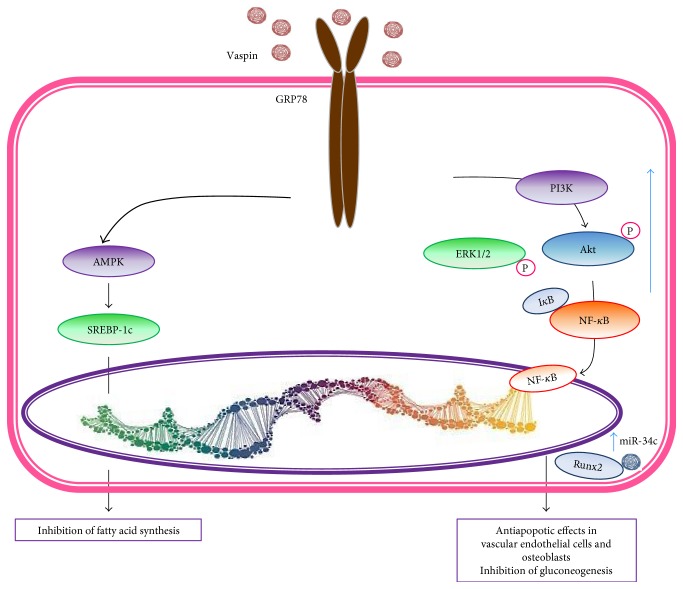
Vaspin signaling. Vaspin binds its receptor, glucose-regulated protein 78 (GRP78) GRP78, and activates the expression of Bcl-2 and downregulates that of Bax. Moreover, vaspin stimulates the PI3K signaling pathway with a specific phosphorylation of ERK and AKT. Vaspin has antiapoptotic effects in vascular endothelial cells and human osteoblasts.

Vaspin (visceral AT-derived serine protease inhibitor) has been identified as an adipokine that is expressed predominantly in visceral AT. It has showed that vaspin could attenuate the osteogenic differentiation in the preosteoblast cell line MC3T3-E1 by the increment of microRNA-34c and its binding to Runx2. Runx2 is a transcription factor that modulates the expression of multiple bone-related genes (type I collagen, osteocalcin, and bone sialoprotein) through PI3K-Akt and ERK signaling pathway (Figure 9) [201]. In vascular smooth muscle cells inflammation, vaspin exerts an anti-inflammatory effect by inhibiting the TNF-*α*-induced ICAM-1 expression, reactive oxygen species, proinflammatory adipokines (resistin and leptin), and TNF-*α* in murine WAT, through decrease phosphorylation of NF-*k*B and PKC*θ* (Figure 9) [111].

It has been demonstrated that cartilage, synovium, meniscus, infrapatellar fat pad, and osteophyte from OA patients expressed vaspin gene; the protein is only expressed by the superficial zone of OA patient's cartilage, the clusters of synovial cells, and the transitional layer of osteophytes between cartilage and fibrous tissues. Regarding to circulating vaspin levels, the serum concentration was reduced in OA patients compared to healthy controls and serum vaspin levels from OA patients surpass those in the paired SF. Serum or SF vaspin was not related to age and BMI. However, vaspin levels were higher in males compared with females, but with no statistical significance (Figure 3(h)) [130]. This suggests a potential protective role of vaspin in OA (Figure 1(b)).

### 3.9. Nesfatin-1

Nesfatin-1 is expressed by chondrocytes, osteophytes, and synovial tissue of knee OA. It induces the expression of COX-2 and the release of IL-8, IL-6, and MIP-1*α*, in human primary chondrocytes from OA patients [202]. Nesfatin levels in OA serum are significantly higher, as compared to SF samples and serum from healthy controls (Figure 3(i)). Significant correlation is found between serum nesfatin-1 and hsCRP levels in OA patients and synovial nesfatin-1 and IL-18 levels. Thus, nesfatin-1, hsCRP, and IL-18 could be considered as biomarkers to determine the knee OA progression [203]. Furthermore, Zhang et al. determined that serum and SF nesfatin-1 levels were both significantly associated with OA severity (Figure 1(b)) [204].

## 4. Therapeutic Perspectives

Even though there is strong evidence of the relationship between adipokines and OA, there is no therapeutic proposal regarding the regulation of the production or function of the former. Leptin activity may be detrimental in some pathological conditions such as enhancement of undesired immune responses in chronic inflammatory diseases, autoimmune diseases, cancer, elevated blood pressure, and certain cardiovascular pathologies. The recent development of monopegylated superactive leptin muteins exhibiting antagonistic properties and other leptin-action-blocking peptides, proteins, neutralizing circulating monoclonal antileptin or leptin receptor antibodies, and nanobodies (variable domains of the *Camelidae* family heavy-chain antibodies) opens a variety new perspectives for their use in research, and finally, as promising therapeutic tools for the treatment of the inflammatory pathologies described above by blocking CD4^+^ T lymphocyte activation and proliferation, cytokine secretion, phagocytosis, regulation of the hypothalamic-pituitary-adrenal axis, reproduction, and angiogenesis and by inducing expansion of naturally occurring Foxp3^+^CD4^+^CD25^+^ regulatory T (Treg) cells [205–207].

In addition, to explore the conversion of the WAT to uncoupling protein-1- (UCP-1-) expressing adipocytes with thermogenic capacity (beige or brown tissue (BAT), or the activation of BAT) is also a fertile ground in research for the development of novel therapeutic technologies. The brown and beige adipocytes have the capacity to counteract metabolic disease, including obesity and type 2 diabetes and obesity. It has been reported that the levels of peroxisome proliferation-activated receptor-*γ* coactivator 1*α* (PGC-1*α*) are increased in muscle by exercise stimulating the secretion of a membrane protein fibronectin type III domain-containing 5 (FNDC5), a potential transcriptional target of PGC-1*α*. FNDC5 is proteolytically cleaved to release a shorter peptide named irisin. Thus, irisin is an exercise-induced myokine that acts on WAT to stimulate UCP-1 expression to increase levels of brown-like fat. Hence, irisin could be therapeutic for human inflammatory diseases [208–210].

It is important to highlight that the risk of OA can be decreased by losing weight and in consequence fat mass (WAT); it has been estimated that if an individual decrease body mass index (fat mass) by 2 units (approximately 5 kg), the risk of developing knee OA would decrease by over 50% [211]. In a study that assessed the impact of a combined pain coping skills training and behavioral weight management in 169 overweight and obese patients with knee OA, a reduction of leptin levels after 24 weeks of the program when compared with baselines levels was observed. This decline was clearly mediated by weight change. However, the authors did not find differences regarding adiponectin [212]. Also, among a cohort of patients who underwent bariatric surgery and had symptomatic OA, leptin serum levels were elevated. After the surgery, both leptin levels and pain fell, suggesting that the fall of leptin could contribute to the knee pain relief [213].

Finally, it is not preposterous to consider the possibility of performing autologous subcutaneous adipose tissue transplants to improve adipose tissue metabolism and reduce insulin resistance and consequently the synthesis and production of adipokines [214].

## 5. Conclusion

**Figure 10:**
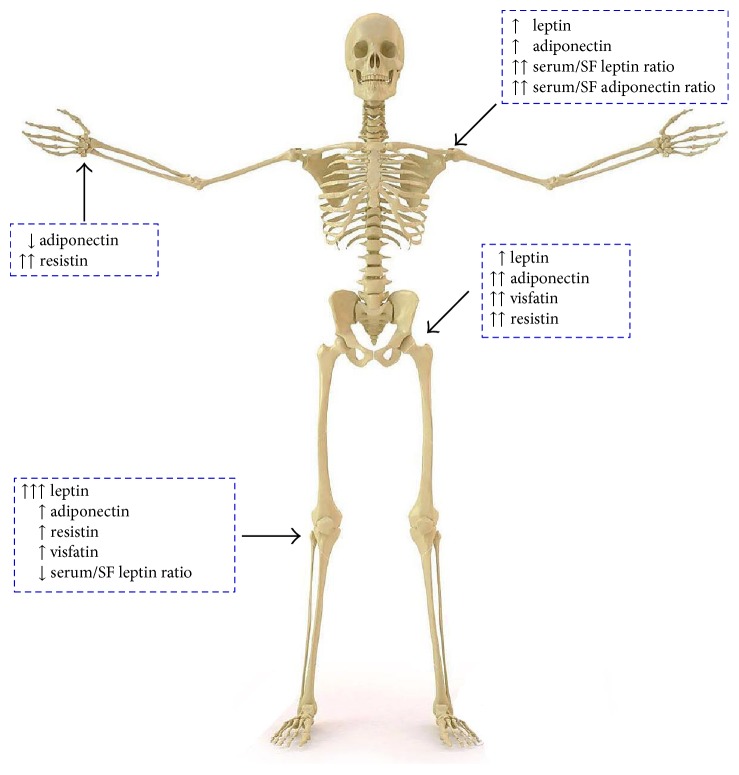
Expression of adipokines in large and small joints. Hand: Different studies have demonstrated that adiponectin may have a protective role in knee OA and it may be related to erosive hand OA [163, 164, 166]. Choe et al. showed that serum levels of resistin correlate with radiographic changes, in specific with subchondral erosions but with no pain [173]. Knee: there are multiple studies that show a higher level of leptin in synovial fluid and serum, and this correlates with the damage of the joint. The higher SF levels are thought to be related to the presence of infrapatellar fat (IFP) pad that produces leptin locally [215, 216]. Resistin and visfatin are produced by the IFP, and their levels correlate with joint damage and the levels of IL-6. Resistin also correlates with menisci damage. Hip: even though the hip and knee are under similar stress conditions, the clinical features and the adipocytokine profile are different, with lower levels of leptin and higher adiponectin, resistin, and visfatin levels within the joint. Only the levels of visfatin correlate with hip pain [140]. Shoulder: the leptin and adiponectin levels correlate with joint damage, but appear to have a different profile of adipocytokines in shoulder, with lower SF and serum levels of leptin and adiponectin, but a higher serum/SF ratio for both, especially adiponectin [168].

Adipokines are synthesized and upregulated by adipocytes as well as chondrocytes and other cell types from joints with OA. The immunomodulatory effects of adipokines imply altered local but also systemic inflammation. Up to date, the best-studied adipokines are adiponectin, leptin, visfatin, and resistin (Figure 10), although new adipokines have been added to the list. The presence of these adipokines has been recognized in the synovium, infrapatellar fat pad, and chondrocytes of patients with OA. Further research is still needed to understand the role of each of the adipokines in the development and progression of OA as well as how it is related to obesity and metabolic factors. Right now, it is still unknown if important changes in adipokine levels induced by a drug or chance of lifestyle will truly impact cartilage loss or any other important outcome in OA. However, future approaches to antagonize local specific target adipokines in OA, but with minimum systemic adverse effects, are warranted.

## Figures and Tables

**Table 1:** Effects of adipokines on OA pathogenesis.

	Cartilage	Bone	Proteases	Cytokines	Inflammation
Leptin	↓ chondrocyte proliferation ↓ proteoglycan synthesis ↑ type I collagen synthesis	↑ osteoblast proliferation ↑ ossification ↑ alkaline phosphatase ↑ osteocalcin	↑ MMP1, MMP-13 ↑ MMP3, MMP2/9 ↑ ADAMTS-4, ADAMTS-5, ADAMTS-9 ↑ cysteine proteases	↑ IL-1*β*, IL-6 ↑ TNF-*α*, TGF-*β*1, IGF-1 ↑ IL-8, GRO, MCP1 ↓ FGF	↑ NOS2, iNOS ↑ PGE_2_, COX-2 ↑ uCTX-II ↑ sCOMP

Adiponectin	↑ chondrocyte proliferation ↑ proteoglycan synthesis ↑ type II collagen synthesis ↓ matrix mineralization	↑ osteoblast proliferation ↑ osteoclast differentiation ↑ RANKL ↓ OPG	↑ MMP1 ↑ MMP3, MMP2/9 ↑ TIMP1, TIMP2 ↓ MMP13	↓ IL-1*β*, IL-6 ↓ IL-8, MCP1, CCL2 ↓ VEGF	↓ NOS2, iNOS ↓ PGE_2_ ↓ uCTX-II, sCOMP ↓ VCAM-1, ICAM-1

Resistin	↓ proteoglycan synthesis ↓ type II collagen synthesis	↑ osteoblast proliferation ↑ osteoclast differentiation	↑ MMP1, MMP-13 ↑ ADAMTS-4	↑ IL-1*β*, IL-6 ↑ TNF-*α* ↑ CCL2, CX3CL1 ↑ BMP2	↑ PGE_2_ ↑ hsCRP

Visfatin	↓ chondrocyte phenotype ↓ proteoglycan synthesis ↓ type II collagen synthesis	↑ osteoblast proliferation ↓ osteoclast differentiation	↑ MMP-13 ↑ MMP3 ↑ ADAMTS-5	↑ IL-1*β*, IL-6 ↑ TNF-*α* ↑ IL-8, MCP1 ↑ NGF	↑ NO ↑ PGE_2_, hsCRP ↑ uCTX-II ↑ AGG1/AGG2

ADAMTS: a disintegrin and metalloproteinase with thrombospondin motifs; AGG: aggrecan; BMP: bone morphogenetic protein; CCL2: chemokine C-C motif ligand 2; COMP: cartilage oligomeric matrix protein; COX-2: cyclooxygenase-2; CX3CL1: chemokine (C-X3-C motif) ligand 1; FGF: fibroblast growth factor; GRO: growth-related oncogene; hsCRP: high-sensitivity C-reactive protein; ICAM-1: intercellular adhesion molecule-1; IGF-1: insulin-like growth factor-1; IL: interleukin; iNOS: inducible nitric oxide synthase; MCP-1: monocyte chemoattractant protein-1; MMP: metalloproteinases; NGF: neuronal growth factor; NO: nitric oxide; NOS2: type 2 nitric oxide synthase; OPG: osteoprotegerin; PGE_2_: prostaglandin E_2_; RANKL: receptor activator of nuclear factor-kappa B ligand; sCOMP: synovial cartilage oligomeric matrix protein; TIMP: tissue inhibitor of metalloproteinases; TGF-*β*: transforming growth factor-*β*; TNF-*α*: tumor necrosis factor-alpha; uCTXII: urine C terminal telopeptide of type II collagen; VCAM-1: vascular cell adhesion molecule-1; VEGF: vascular endothelial growth factor.

## Abbreviations

ADAMST: Disintegrin and metalloproteinase with thrombospondin motifs
ADSF: AT-specific secretory factor
AKT: Protein kinase B
AMPK: 5′-adenosine monophosphate-activated protein kinase
AT: Adipose tissue
BMI: Body mass index
XCP1: C/EBP-epsilon-regulated myeloid-specific secreted cysteine-rich protein
CaMKII: Calmodulin-dependent protein kinase II
CAT: Catalase
CAP-1: Adenylyl cyclase-associated protein 1
CCL-2: CC-chemokine ligand 2
CCRL2: Chemokine C-C motif receptor
CD: Cluster differentiation
CKMLR1: Chemokine-like receptor ChemR23
COX-2: Cyclooxygenase-2
CRP: C-reactive protein
CTX-II: C-telopeptide of type II collagen
ELAM-1: Endothelial-leukocyte adhesion molecule 1
ERK: Extracellular signal-regulated kinases
FGFR2: Fibroblast growth factor receptor 2
G-CSF: Granulocyte colony-stimulating factor
gp130: Glycoprotein 130
GRB2: Growth factor receptor-bound protein 2
GRO: Growth-related oncogene
GRP78: Glucose-regulated protein 78
GSHPx: Glutathione peroxidase
HLA-DR: Human leukocyte antigen-DR
hsCRP: High-sensitivity C-reactive protein
ICAM-1: Intercellular adhesion molecule-1
IFP: Infrapatellar fad pad
IGR-1: Insulin-like growth factor 1 receptor
IL: Interleukin
JAK: Janus kinases
JNK: Jun N-terminal kinases
LCN2: Lipocalin 2
LIFR: Leukocyte inhibitory factor receptor
LK: Liver kinase
LPS: Lipopolysaccharide
MAPK: Mitogen-activated protein kinases
MMP: Matrix metalloproteinase
NAD: Nicotinamide adenine dinucleotide
NAmPRTase or Nampt: Nicotinamide phosphoribosyltransferase
NF-*k*B: Nuclear factor-kappa B
NGF: Nerve growth factor
NKs: Natural killer cells
NO: Nitric oxide
NUCB2: Nucleobindin 2
OA: Osteoarthritis
OASF: OA synovial fibroblasts
PBEF1: Pre-B cell colony-enhancing factor 1
PGE_2_: Prostaglandin E_2_
PI3K: Phosphatidylinositol 3 kinase
PKC: Protein kinase C
PPAR: Peroxisome proliferator-activated receptors
RARRES2: Retinoic acid receptor responder protein 2
ROR1: Tyrosine kinase-like orphan receptor-1
SHP2: Src-homology 2 domain-containing phosphatase 2
SF: Synovial fluid
siRNA: Small interfering RNA
SOD: Superoxide dismutase
SOX9: Sex determining region-box 9
STAT: Signal transducers and activators of transcription
TIG2: Tazarotene-induced gene 2 protein
TIMP: Tissue inhibitor of metalloproteinases
TNF-*α*: Tumor necrosis factor-*α*
TLR: Toll-like receptor
VCAM-1: Vascular cell adhesion molecule-1
VEGF: Vascular endothelial growth factor
WAT: White adipose tissue.
